# Antimicrobial Activity and Antibiofilm Potential of Coenzyme Q_0_ against *Salmonella* Typhimurium

**DOI:** 10.3390/foods10061211

**Published:** 2021-05-27

**Authors:** Zhuokai Yang, Xiaoyu Ma, Yan Li, Huidong Xu, Xinyi Han, Ruixia Wang, Pengyu Zhao, Ziyi Li, Chao Shi

**Affiliations:** 1 College of Food Science and Engineering, Northwest A&F University, Yangling 712100, China; zhuokaiy@student.unimelb.edu.au (Z.Y.); maxiaoyu2020@163.com (X.M.); hanxinyi@nwafu.edu.cn (X.H.); ruixiawang@nwsuaf.edu.cn (R.W.); zhaopengyu@nwsuaf.edu (P.Z.); 2 College of Innovation and Experiment, Northwest A&F University, Yangling 712100, China; liyan2727@nwafu.edu.cn (Y.L.); xhd2018015295@nwafu.edu.cn (H.X.); yunshanglzy@nwafu.edu.cn (Z.L.)

**Keywords:** coenzyme Q_0_, *Salmonella* Typhimurium, cell membrane, biofilm, raw chicken

## Abstract

Coenzyme Q_0_ (CoQ_0_) has anti-inflammatory and anti-tumor effects; however, the antimicrobial and antibiofilm activities of CoQ_0_ against *Salmonella enterica* serovar Typhimurium are unknown. Thus, we investigated the bacteriostatic and antibiofilm activities, along with the underlying mechanism, of CoQ_0_ against *S*. Typhimurium. The minimum inhibitory concentration (MIC) of CoQ_0_ against *S. enterica* serovars Typhimurium was 0.1–0.2 mg/mL (549–1098 µM), and CoQ_0_ at MIC and 2MIC decreased viable *S*. Typhimurium counts below detectable limits within 6 and 4 h, respectively. CoQ_0_ at 20MIC (4 mg/mL) reduced *S*. Typhimurium on raw chicken by 1.5 log CFU/cm^3^ within 6 h. CoQ_0_ effectively disrupted cell membrane integrity and induced morphological changes in the cell, resulting in hyperpolarization, decreased intracellular ATP concentrations, and cellular constituents leakage. Biofilm-associated *S*. Typhimurium cells were killed by CoQ_0_ treatment. These findings suggest that CoQ_0_ could be applied as a natural antibacterial substance for use against *S*. Typhimurium by the food industry.

## 1. Introduction

The genus *Salmonella* comprises several species and numerous sub-species and serovars, all of which are foodborne opportunistic bacterial pathogens that can be transmitted between humans and animals [1]. These Gram-negative, rod-shaped, flagellated bacteria are widely distributed and are common contaminants of foods, especially meat, egg, and dairy products, which are some of the main sources of foodborne illness [2]. *Salmonella* infection can cause gastroenteritis, typhoid fever, sepsis, and other syndromes, the combined effects of which have the greatest impact on *Salmonella* mortality rates [3]. Worldwide, an estimated 94 million disease cases and 155,000 deaths have been attributed to *Salmonella* infection in the last decade [4].

Biofilms are aggregates of bacterial cells with a certain spatial structure. Within these aggregates, bacteria proliferate and secrete an extracellular matrix that helps to anchor the cells to each other and, often, to a surface [5]. *Salmonella* readily form biofilms on multiple surface types found in the food production chain, including plastics, glass, stainless steel, wood, and rubber [6]. When a biofilm reaches the stage of irreversible adhesion, it cannot be removed by gentle cleaning [7]. In addition, compared with planktonic cells, biofilm-associated *Salmonella* demonstrate increased pathogenicity, including enhanced adaptability and resistance to various antibiotics, physical and chemical disinfectants, environmental stress, and the human immune response [8].

Preventing *Salmonella* contamination of food products, corrosion of food production facilities, and human infections is of significance to public health worldwide [9]. Various chemical preservatives are commonly used in food processing, production, and storage to kill *Salmonella* and other pathogenic microorganisms in food; however, the extensive use of chemical preservatives has led to increased resistance among pathogenic microorganisms [10]. Further, traditional methods of removing *Salmonella* biofilms have limitations: disinfectants used in the food industry have significant scavenging effects on biofilm but can produce carcinogenic by-products [11]. In recent years, there has been increased interest in plant-based antimicrobials, which are widely distributed in nature and have a variety of physiological activities. More importantly, these compounds can be used as highly effective broad-spectrum bacteriostatic agents in place of chemical preservatives to control foodborne pathogens [12]. *Antrodia camphorata* is a wild fungus with significant utility in the production of plant-based antimicrobials. It contains high levels of coenzyme Q_0_ (CoQ_0_; 2,3-dimethoxy-5methyl-1,4-benzoquinone; molecular weight, 182.1733, Figure 1), an anthraquinone substance with anti-cancer and anti-inflammatory activities [13,14,15,16]. CoQ_0_ has also been shown to inhibit and kill important human pathogens such as *Cronobacter sakazakii*, *Vibrio parahaemolyticus*, *Listeria monocytogenes*, and *Staphylococcus aureus*. However, there is little information on the effects of CoQ_0_ on *Salmonella* or its ability to remove biofilms [17,18,19].

In this study, the effects of CoQ_0_ on *S.* Typhimurium were investigated to discern the mechanism of CoQ_0_ inhibition/killing of planktonic bacteria, and to assess its ability to eliminate *S.* Typhimurium biofilms. Based on the minimum inhibitory concentration (MIC) and minimum bactericidal concentration (MBC) of CoQ_0_ against *S.* Typhimurium, its effects on bacterial survival, intracellular ATP concentration, cell membrane potential, intracellular compound leakage, cell membrane permeability, and cell morphology were investigated. We also assessed the ability of CoQ_0_ to eliminate *S.* Typhimurium biofilms.

## 2. Material and Methods

### 2.1. Reagents

Coenzyme Q_0_ (HPLC ≥ 99%, CAS 605-94-7) was purchased from J&K Scientific Co. (Beijing, China) and was dissolved in dimethyl sulfoxide (DMSO). The final concentration of DMSO in all sample solutions was 1% (*v*/*v*), which has no apparent effect on the growth of *S.* Typhimurium. Luria–Bertani (LB) and buffered peptone water (BPW) were purchased from Land Bridge Technology Co. (Beijing, China). All other chemicals and reagents were of analytical grade.

### 2.2. Bacterial Strains and Growth Conditions

*Salmonella enterica* subsp. *enterica* serovar Typhimurium ATCC 14028, and *S.* Typhimurium AS1.1174 were obtained from the American Type Culture Collection (ATCC, Manassas, VA, USA). Five other *S.* Typhimurium strains (Table 1) were isolated from raw chicken and beef by our experimental team. All seven *S.* Typhimurium strains were used in MIC and MBC assays, while *S.* Typhimurium ATCC 14028 was used in all subsequent experiments.

*S.* Typhimurium strains were stored at −80 °C before being cultured at 37 °C for 18 h on LB agar. To obtain fresh overnight cultures, stored isolates were inoculated into LB broth medium in 50-mL conical flasks and incubated at 37 °C for 12 h with shaking at 130 rpm. The cultures were then centrifuged (8000× *g*, 5 min, 4 °C), washed twice with sterile phosphate-buffered saline (PBS; pH 7.2), and resuspended in LB broth to an optical density at 600 nm (OD_600_) of 0.5, which was equivalent to a cell concentration of approximately 10^9^ colony-forming units (CFU/mL). 

### 2.3. Determination of MIC and MBC Values

The MIC of CoQ_0_ against the different *S.* Typhimurium strains was determined using the broth microdilution method described by Guo et al. [17], with some modifications. Briefly, bacteria were diluted to a concentration of 5 × 10^5^ CFU/mL in LB broth. Equivalent volumes (100 μL) of prepared bacterial suspension and CoQ_0_ solution were added to the wells of 96-well microtiter plates to achieve final CoQ_0_ concentrations of 0.8, 0.4, 0.2, 0.1, 0.05, and 0 (control) mg/mL. LB broth containing 1% (v/v) DMSO was used as the negative control. The OD_600_ of each well was measured using a microtiter plate reader (Model 680; Bio-Rad, Saint Louis, MO, USA), and then plates were incubated at 37 °C for 24 h. OD_600_ measurements were repeated at the end of the incubation period. The MIC of CoQ_0_ was defined as the lowest concentration that resulted in a change in OD_600_ (final OD_600_ value minus initial OD_600_ value) of ≤ 0.05. For MBC determinations, 100 μL of bacterial suspension from each well was plated onto an LB agar plate. Following incubation for 48 h at 37 °C, the MBC was defined as the lowest antimicrobial concentration that did not allow any bacterial growth.

### 2.4. Inactivation of S. Typhimurium by CoQ_0_ in LB

The bactericidal activity of CoQ_0_ against *S.* Typhimurium ATCC 14028 was tested using the plate count method described by Kang and Song [20], with slight modification. *S.* Typhimurium was first diluted to a concentration of 1 × 10^8^ CFU/mL. Equal volumes (10 mL) of LB broth and CoQ_0_ were mixed in 15-mL test tubes to achieve final CoQ_0_ concentrations of 0, 0.1 (1/2MIC), 0.2 (MIC), and 0.4 mg/mL (2MIC). A 100-μL aliquot of *S.* Typhimurium suspension was then added to each test tube and cultured at 37 °C. Samples were collected at 0, 1, 2, 4, 6, 9, and 12 h post-inoculation, serially diluted in PBS (pH 7.2), plated on LB agar, and incubated at 37 °C for 18 h. Following incubation, the numbers of *S.* Typhimurium colonies were counted.

### 2.5. Antimicrobial Effect of CoQ_0_ on Inoculated S. Typhimurium on Raw Chicken

The experimental method of Roh et al. [21] was used to test the antibacterial effect of CoQ_0_ on *S.* Typhimurium on raw chicken, with slight modification. Boneless, skinless chicken breasts were purchased by online shopping (Jindong, China). The chicken breasts were first cut into uniform pieces (1.5 × 1.5 × 1 cm) and placed in a sterile sealed bag for irradiation. After 2 s of 4 kGy irradiation, the background bacteria on raw chicken were completely removed, then raw chicken was stored in the refrigerator at −20 °C until use. *S.* Typhimurium was diluted to a concentration of 1 × 10^9^ CFU/mL, then the bacteria solution was inoculated on the surface of chicken breast meat (20 μL per piece), and dried for 30 min. BPW broth (10 mL), DMSO (1%), and CoQ_0_ were mixed in aseptic sealed bags to achieve final CoQ_0_ concentrations of 0 (Control), 1 (5MIC), 2 (10MIC), and 4 mg/mL (20MIC). The chicken samples were added to each sterile sealed bag and cultured for 30 min, and then the chicken samples were taken out and stored in the refrigerator at 12 °C. At 0, 3, and 6 h, the chicken samples were taken out and shaken in a homogenizer in 0.1% BPW for 3 min, collection fluid (1 mL) serially diluted in PBS (pH 7.2), plated on LB agar, and incubated at 37 °C for 12 h. Following incubation, the numbers of *S.* Typhimurium colonies were counted.

### 2.6. Determination of the Antibacterial Mechanism of CoQ_0_ against Planktonic S. Typhimurium Cells

#### 2.6.1. Membrane Potential

The membrane potential of *S.* Typhimurium ATCC 14028 cells following CoQ_0_ treatment was determined as described by Shi et al. [22]. Briefly, *S.* Typhimurium diluted in PBS (OD_600_ = 0.5) was seeded into black 96-well microtiter plates, and the same volume of PBS was also seeded into black 96-well microtiter plates as background. Following incubation, 1 μM bis-(1,3-dibutyl barbituric acid) trimethine oxonol (DiBAC_4_(3); Molecular Probes, Eugene, OR, USA) was used as a membrane potential-sensitive fluorescent probe and added to the *S.* Typhimurium suspensions and PBS for 30 min at 37 °C in the dark. CoQ_0_ was then added to each well to achieve final concentrations equivalent to 0 (control), MIC, and 2MIC. 

Following a further incubation for 5 min on the bench, fluorescence was measured using a fluorescence microplate reader (Spectra Max M2; Molecular Devices, San Jose, CA, USA) at excitation and emission wavelengths of 492 and 515 nm, respectively. Membrane potential was illustrated in terms of relative fluorescence units (RFUs; RFU = (FU_treatment_ − FU_background_) − (FU_control_ − FU_background_)), with three independent replicates included in each experiment.

#### 2.6.2. Intracellular ATP

Intracellular ATP concentrations were determined as described by Fei et al. [23], with some modification. Briefly, *S.* Typhimurium ATCC 14028 resuspended in PBS to ~10^9^ CFU/mL was mixed with CoQ_0_ at concentrations equivalent to 0 (control), MIC, and 2MIC, and incubated for 30 min in 37 °C. Following incubation, all samples were ultrasonicated on ice. Following ultrasonication, *S.* Typhimurium suspensions were heated at 100 °C for 180 s and then centrifuged for 5 min (5000× *g*, 4 °C). The resulting supernatants were collected and stored on ice for measurement of intracellular ATP concentration.

ATP concentrations were measured using an ATP assay kit as per the manufacturer’s instructions (Beyotime Bioengineering Institute, Shanghai, China). Briefly, 100 μL aliquots of the extracted ATP-containing supernatants and the ATP standard solutions were added to white 96-well microtiter plates. An equal volume of ATP detection reagent was then added to each well. The luminescence of each well was determined using a multimode microplate reader (Spectra Max M2; Molecular Devices). The intracellular ATP concentrations of the samples were calculated from a standard curve generated from the luminescence values of the ATP standard solutions. Three independent replicates of each experiment were performed.

#### 2.6.3. Release of Cellular Constituents

The release of cellular constituents from *S.* Typhimurium ATCC 14028 was measured as described by Li et al. [24]. *S.* Typhimurium resuspended in PBS to an OD_600_ of 0.5 (30 mL) was added to three 50-mL centrifuge tubes, and 30-mL PBS was added to the other three 50-mL centrifuge tubes as background. CoQ_0_ was added to each tube to achieve final concentrations equivalent to 0 (control), MIC, and 2MIC. Following vortex oscillation, the samples were incubated at 37 °C for 240 min. Each sample was then centrifuged (5000× *g*, 4 °C, 15 min) and filtered through a 0.22-μm filter membrane. The absorbance measured at 260 nm using a microplate reader (Spectra Max M2; Molecular Devices) indicated the concentration of the constituents released from the cells (Absorbance = Abs_treatment_ − Abs_background_).

#### 2.6.4. Membrane Integrity

The effect of CoQ_0_ on the integrity of *S.* Typhimurium cell membranes was assessed using two nucleic acid dyes, SYTO 9 (green) and propidium iodide (PI, red), as described previously [25], with minor modification. Briefly, *S.* Typhimurium ATCC 14028 cells were washed three times in 0.85% (w/v) NaCl solution before being resuspended in 0.85% (*w/v*) NaCl solution to a concentration of ~1 × 10^9^ CFU/mL. CoQ_0_ was added to the bacterial cell suspensions at concentrations equivalent to 0, MIC, or 2MIC and incubated at 37 °C for 30 min. Following incubation, the suspensions were centrifuged at 10,000× *g* for 2 min and the resulting bacterial pellets resuspended in 1 mL of 0.85% (*w/v*) NaCl solution. The resuspended cells were then mixed with 3 μL of fluorescent marker solution, consisting of 1.5 μL of SYTO 9 and 1.5 μL of PI, and incubated at room temperature for 5 min in the dark. Finally, one drop of each sample was placed onto a glass slide, covered with a coverslip, and observed by confocal laser scanning microscopy (CLSM; A1; Nikon, Tokyo, Japan) at 800× magnification.

#### 2.6.5. Bacterial Morphology

The morphology of *S.* Typhimurium ATCC 14028 was observed using field emission scanning electron microscopy (FESEM), as described by Shi et al. [22]. Briefly, *S.* Typhimurium ATCC 14028 cells (~1 × 10^9^ CFU/mL) were treated with CoQ_0_ at concentrations equivalent to 0, MIC, 2MIC, and 4MIC and incubated at 37 °C for 2 or 4 h. Cells were then harvested by centrifugation (5000× *g*, 5 min, 4 °C), rinsed twice with PBS, and resuspended in 2.5% (*v/v*) glutaraldehyde at 4 °C for initial fixation. The cells were then washed with PBS and sterile water before being dehydrated gradually in a graded water–ethanol series (30, 50, 70, 80, 90, and 100% ethanol) for 10 min. Finally, the samples were dried, gold sputtered, and observed using a field-emission scanning electron microscope (S-4800; Hitachi, Tokyo, Japan) at 20,000× magnification. In the 20,000× magnification field of view, areas with less background impurities and low-density cells were searched, and then *S.* Typhimurium ATCC 14028 cells with typical characteristics under the treatment of the corresponding CoQ_0_ concentration were identified and selected to make SEM images.

### 2.7. CoQ_0_ Inactivation of S. Typhimurium Biofilms on Stainless Steel Surfaces

The ability of CoQ_0_ to inactivate biofilm-associated *S.* Typhimurium ATCC 14028 on stainless steel surfaces was examined as described by Zheng et al. [26], with slight modification. *S.* Typhimurium ATCC 14028 suspension was centrifuged (4000× *g*, 15 min, 4 °C) and resuspended in PBS to achieve a concentration of 7.0 log CFU/mL. To encourage biofilm formation, 30-mL aliquots of *S.* Typhimurium ATCC 14028 were added to separate sterile 50-mL test tubes containing a sterile stainless steel sheet (5 cm × 2 cm) and incubated at 25 °C for 72 h.

Following biofilm formation, the sheets were removed from the test tubes using sterile forceps, soaked in 400 mL of sterile distilled water at 25 °C for 15 s, and then rinsed in 400 mL of sterile distilled water with gentle agitation for 5 s to remove weakly adherent cells. The stainless steel sheets were then transferred to sterile tubes containing 30 mL of PBS with/without CoQ_0_ (0, 5MIC, 8MIC, or 10MIC) and incubated at 25 °C for 0, 30, 60, 90, 120, or 180 min. At each sampling point, stainless steel sheets were rinsed in 400 mL of sterile distilled water with agitation for 15 s and then in 400 mL of sterile distilled water for 5 s, before being placed in sterile 50-mL centrifuge tubes containing 3 g of glass beads (G8772, 425–600 μm; Sigma-Aldrich, St. Louis, MO, USA) and 30 mL of sterile PBS, and then vortexed for 5 min. Finally, the bacterial suspensions were plated onto LB plates and incubated at 37 °C for 24 h.

### 2.8. Statistical Analysis

The whole experiment of each assay was repeated three times independently. SPSS statistical analysis software v. 19.0 (SPSS Inc., Chicago, IL, USA) was used to process the data, and the results were expressed as mean ± SD. Differences were analyzed by one-way analysis of variance, and significance was determined using Tukey’s test and least significant difference analysis. Significance was indicated as follows: *, *p* < 0.05; **, *p* < 0.01.

## 3. Results

### 3.1. MICs and MBCs

As shown in Table 1, the MIC of CoQ_0_ against *S.* Typhimurium ATCC 14028, *S.* Typhimurium AS1.1174, and food isolates 22, 64, 80, and 98 was 0.2 mg/mL (1098 µM), and the MIC of CoQ_0_ against food isolate 63 was 0.1 mg/mL (549 µM). The MBC values of all tested strains in this study were identical to the MIC values. *S.* Typhimurium ATCC 14028 was selected as a model organism for use in all subsequent experiments.

### 3.2. Inactivation of S. Typhimurium by CoQ_0_ in LB

Based on the results of MIC assays, various concentrations of CoQ_0_ were used to investigate its inhibitory effects on *S.* Typhimurium ATCC 14028. As shown in Figure 2, total viable cell counts were initially the same across all treatment groups (~5.8 log CFU/mL). However, cell counts of *S.* Typhimurium ATCC 14028 exposed to CoQ_0_ at 0 and 1/2MIC were 9.1 and 6.9 log CFU/mL at 12 h post-inoculation, respectively, while those of cultures exposed to CoQ_0_ at MIC and 2MIC were below detectable levels at 6 and 4 h post-treatment. 

### 3.3. Antimicrobial Activity of CoQ_0_ on Raw Chicken

The antimicrobial activity of CoQ_0_ on *S.* Typhimurium on raw chicken is shown in Figure 3. The initial number of viable *S.* Typhimurium cells on raw chicken was similar among all treatment groups (~6.7 log CFU/cm^3^). The bacterial count of the control group declined from 6.7 ± 0.1 to 6.5 ± 0.1 log CFU/cm^3^ within 6 h, while the bacterial count of 5MIC and 10MIC groups decreased by 0.4 and 0.7 log CFU/cm^3^, respectively. Simultaneously, the bacterial count of the 20MIC group decreased significantly (*p <* 0.01) to 5.3 ± 0.1 log CFU/cm^3^ within 6 h.

### 3.4. Release of Cell Constituents

Changes in optical density, indicative of the release of intracellular constituents from *S.* Typhimurium ATCC 14028 cells following treatment with CoQ_0_ for 4 h, are shown in Figure 4. There was a significant increase (*p <* 0.01) in the release of cell constituents following treatment with CoQ_0_ at concentrations equivalent to the MIC (OD_260_ = 0.04 ± 0.02) and 2MIC (OD_260_ = 0.1 ± 0.02) compared with the control (OD_260_ = 0.008 ± 0.003). 

### 3.5. Membrane Potential

The effects of CoQ_0_ on the membrane potential of *S.* Typhimurium ATCC 14028 are shown in Figure 5. Hyperpolarization (indicated by negative relative fluorescence values) was observed in *S.* Typhimurium ATCC 14028 samples treated with CoQ_0_ at concentrations equivalent to the MIC and 2MIC, with the degree of hyperpolarization increasing with increases in CoQ_0_ concentration.

### 3.6. Intracellular ATP

Relative luminescence units and ATP concentration were used to establish a standard curve (y = 9249x + 6094.7, R^2^ = 0.9998, the standard curve is not shown) to measure ATP concentration by fluorescence units. As shown in Figure 6, the *S.* Typhimurium ATCC 14028 intracellular ATP concentration was significantly decreased (*p* < 0.01) following CoQ_0_ treatment (MIC, 0.094 ± 0.01 μmol/L; 2MIC, 0.02 ± 0.004 μmol/L) for 30 min compared with the control (0.43 ± 0.02 μmol/L).

### 3.7. Membrane Integrity

SYTO 9, which is a green fluorescent nucleic acid stain that can label all bacteria with intact membranes and damaged membranes, and PI, which is a red fluorescent nucleic acid stain that only penetrates bacteria with damaged membranes, stain bacteria with intact cell membranes with green fluorescence, while bacteria with damaged membranes are stained with red fluorescence to evaluate the injuries of CoQ_0_ to the cell membrane of *S.* Typhimurium ATCC 14028 in CLSM (Figure 7). As shown in Figure 7A, only green fluorescence was observed for control cell cultures. In comparison, increases in the proportion of red versus green fluorescence were observed in cultures treated with increasing concentrations of CoQ_0_ (MIC or 2MIC) (Figure 7B,C).

### 3.8. FESEM-Based Observation of Cell Morphology

SEM images of CoQ_0_-treated and untreated *S.* Typhimurium ATCC 14028 cells are shown in Figure 8. Untreated *S.* Typhimurium ATCC 14028 cells were rod-shaped with a smooth surface (Figure 8A,E), while *S.* Typhimurium cells treated with CoQ_0_ at the MIC appeared wrinkled with a rough surface and notable depressions (Figure 8B,F). When cultures were treated with CoQ_0_ at 2MIC, cell size was dramatically reduced and normal cell morphology was absent (Figure 8C,G). Treatment with CoQ_0_ at 4MIC resulted in membrane rupture (Figure 8D,H). 

### 3.9. Inactivation of S. Typhimurium Biofilms on Stainless Steel Surfaces by CoQ_0_ Treatment

Changes in the number of viable biofilm-associated cells following treatment with CoQ_0_ at 25 °C are shown in Figure 9. At the beginning of treatment, the total number of viable *S.* Typhimurium cells in biofilms on stainless steel surfaces was similar among all treatment groups (~8.1 log CFU/cm^2^). After 3 h, the cell population in untreated biofilms remained at about 8.2 log CFU/cm^2^. However, the number of viable cells in the 5MIC and 8MIC CoQ_0_-treated biofilms decreased significantly to ~6.5 and 5.1 log CFU/cm^2^, respectively, between the 0 and 30 min timepoints before stabilizing. In addition, the number of viable cells in biofilms exposed to 10MIC CoQ_0_ decreased rapidly in the first 30 min, dropping by approximately 4.0 log CFU/cm^2^ within this period, and then continued to decline between 30 and 180 min post-treatment.

## 4. Discussion

In this study, an MIC for CoQ_0_ of 549–1098 µM was observed against *S.* Typhimurium. Previous studies have reported similar inhibitory effects of natural substances against *S.* Typhimurium. For example, Sun, Joy, and Huang reported MICs of carvacrol and thymol against *S.* Typhimurium in the range of 3329–6657 µM, while the MIC of citral against *S.* Typhimurium was ≥ 13,138 µM [27]. In addition, Lin, Liao, and Cui [28] determined an MIC of thyme essential oil against *S.* Typhimurium of 1664 µM, and Buket et al. [10] determined MICs of conventional preservatives, including sodium nitrite, methyl paraben, propyl paraben, potassium sorbate, and sodium benzoate, against *Salmonella* in the range of 110–3624 µM. Therefore, based on our current results, CoQ_0_ appears to have stronger antimicrobial activity against *S.* Typhimurium compared with carvacrol, thymol, thyme essential oil, and citral; besides, the antimicrobial activity of CoQ_0_ is comparable to conventional preservatives.

**Figure 1:**
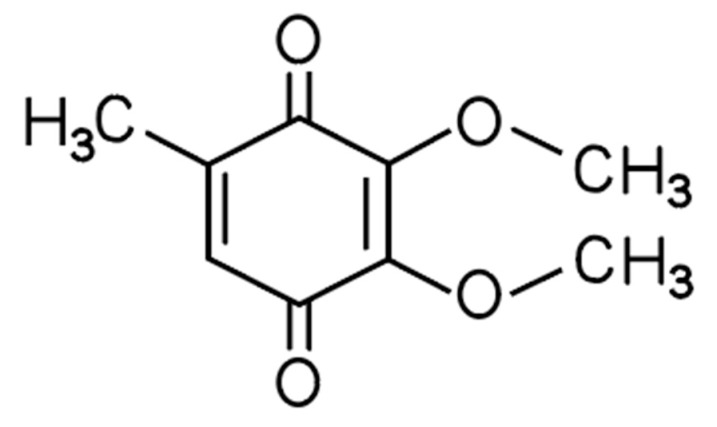
The chemical structure of CoQ_0_.

The inactivation experiment of CoQ_0_ on *S.* Typhimurium in LB showed that CoQ_0_ at concentrations equivalent to the MIC or higher involved significantly decreasing the viable numbers of *S.* Typhimurium cells in LB broth, and confirmed the bactericidal action was irreversible (Figure 1). In comparison, Kang et al. [29] showed that *Shigella flexneri* cells at an initial concentration of 6.4 log CFU/mL decreased to about 1.0 log CFU/mL within 6 h following treatment with gallic acid (GA) at MBC, while incubation with GA at the MIC only resulted in a 1.3-log decline in viable cell counts after 12 h. Prakash et al. [30] found that nanoemulsions of linalool at the MIC reduced *S.* Typhimurium cell counts to below the limits of detection within 2 h; however, after 8 h, the *S.* Typhimurium culture showed renewed growth, suggesting that the effects of linalool nanoemulsions at MIC are reversible. Therefore, in comparison with these other natural compounds, CoQ_0_ shows good inhibitory and germicidal activity against *S.* Typhimurium. Further, the damage caused by CoQ_0_ at concentrations greater than or equal to the MIC is irreversible in *S.* Typhimurium.

**Figure 2:**
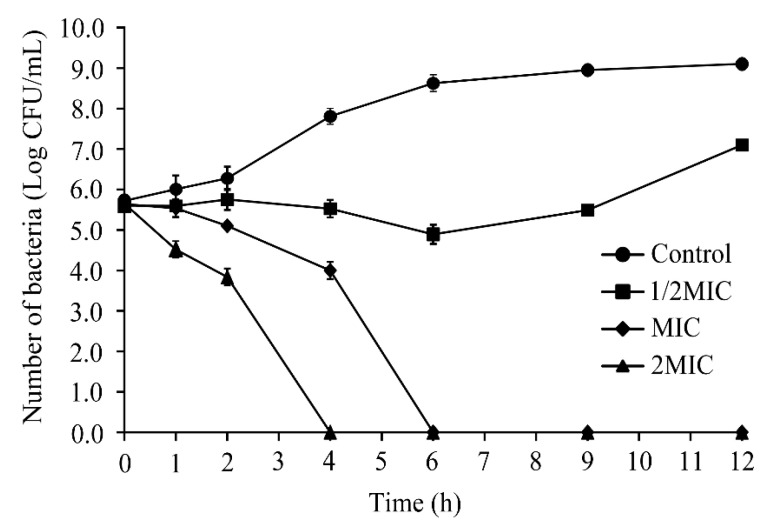
Inactivation of *S.* Typhimurium by CoQ_0_ in LB. (∎) Control, (●) 1/2MIC, (▲) MIC, (♦) 2MIC. Control represents no CoQ_0_ added.

**Figure 3:**
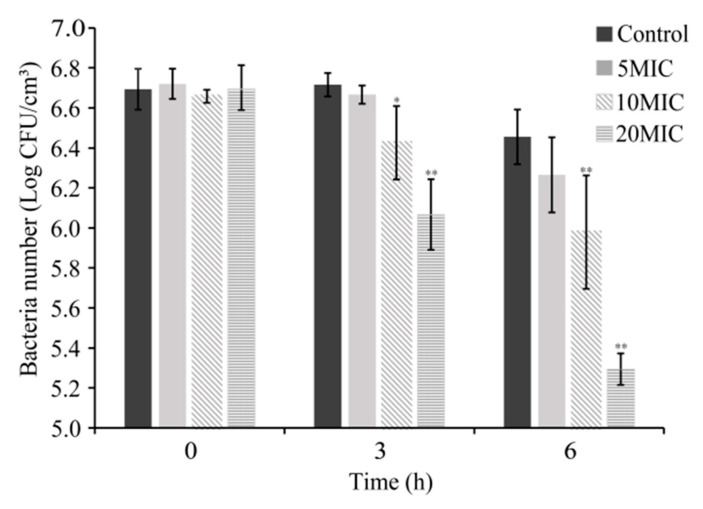
Antimicrobial effect of CoQ_0_ on inoculated *S.* Typhimurium on raw chicken. Bars represent the standard deviation (*n* = 3). Control represents no CoQ_0_ added. * *p* < 0.5 compared with the control, ** *p* < 0.01 compared with the control.

**Figure 4:**
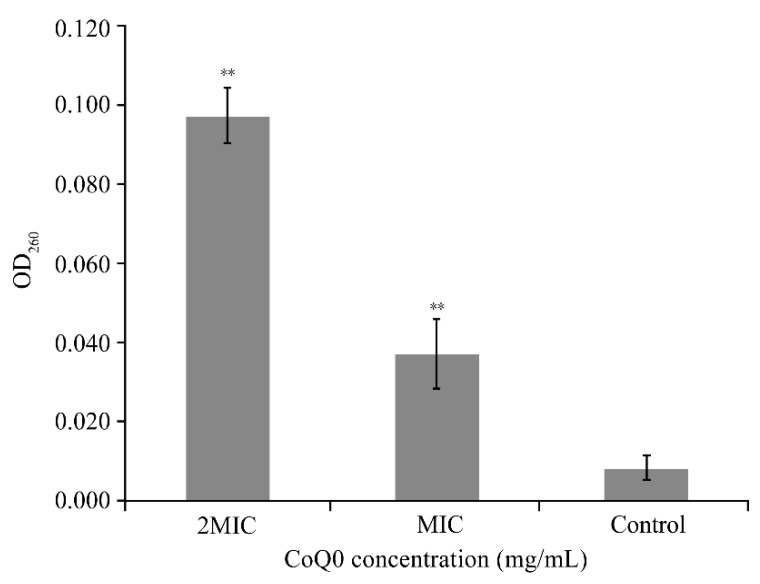
Effect of CoQ_0_ treatment on the release of constituents from *S.* Typhimurium ATCC 14028 cells. Values represent the means of triplicate measurements. Bars represent the standard deviation (*n* = 3). Control represents no CoQ_0_ added. ** *p* < 0.01 compared with the control.

**Figure 5:**
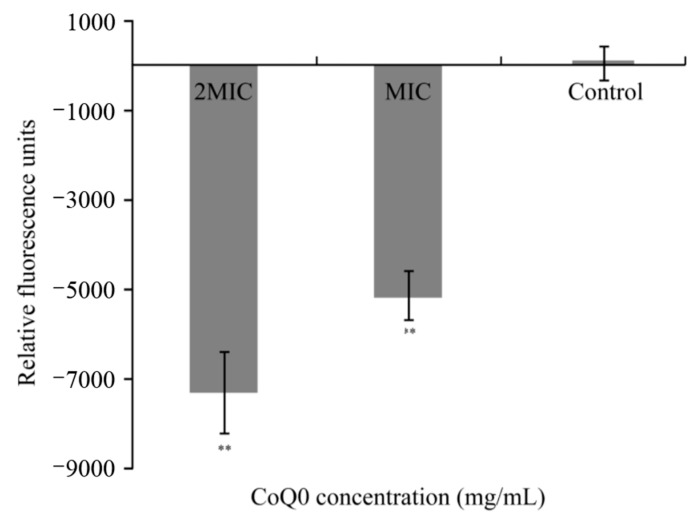
Effects of CoQ_0_ treatment on the membrane potential of *S.* Typhimurium ATCC 14028. Values represent the means of triplicate measurements. Bars represent the standard deviation (*n* = 3). Control represents no CoQ_0_ added. ** *p* < 0.01 compared with the control.

The inhibition experiment of CoQ_0_ on *S.* Typhimurium on raw chicken showed that CoQ_0_ (0.5%) significantly decreased (*p* < 0.01) the viable numbers of *S.* Typhimurium on raw chicken (Figure 2). However, comparing the antibacterial effects of CoQ_0_ in LB broth and on raw chicken (Figure 1 and Figure 2), we found that the antibacterial effect weakened on raw chicken. We speculate that the difference is caused by the temperature change when CoQ_0_ acts (in LB: 37 °C, on raw chicken: 12 °C); besides, the protein and texture of raw chicken reduce the effective contact concentration of *S.* Typhimurium and CoQ_0_. Jang and Rhee reported that the antibacterial effect of caprylic acid on *Cronobacter* spp. (*Enterobacter sakazakii*) is enhanced by the increase in temperature [31], and Guo et al. [18] showed that the antibacterial activity of CoQ_0_ on *Cronobacter sakazakii* decreases as the temperature decreases from 55 to 25 °C. In addition, Jeyakumar et al. [32] found that microalgal fatty acid esters with 1/2MIC concentration can reduce *Listeria monocytogenes* in tryptic soy broth by about 12 log CFU/mL within 9 h, but *L. monocytogenes* on raw chicken only reduced by about 2 log CFU/mL within 9 h. Similarly, Zheng et al. [26] found that CoQ_0_ with a concentration of 100MIC reduced the *Vibro parahaemolyticus* in fresh shrimp from 6.3 to 3.0 log CFU/g within 6 h. Thereby, methods such as synergy with other antibacterial agents, combined with ultrasound, mild heating, or other treatment should be investigated to further enhance the antibacterial effect of CoQ_0_ on *S.* Typhimurium in food.

Our study showed that CoQ_0_ treatment resulted in a significant increase (*p* < 0.01) in the amount of extracellular cell constituents in *S.* Typhimurium cultures. Similarly, Sun et al. [33] examined the effect of anthocyanins on the membrane permeability of *S.* Enteritidis and found that following anthocyanin treatment, cell constituent leakage from *S.* Enteritidis cells increased by 54.1%. Further, Campos et al. [34] showed that cell constituent leakage from *S.* Enteritidis and *S.* Typhimurium cells increased by 51.0 and 50.0%, respectively, following treatment with essential oil extracted from *Mitracarpus frigidus*. Therefore, we speculate that CoQ_0_ causes changes in the permeability of the *S.* Typhimurium cell membrane, resulting in intracellular cell constituent leakage and inhibiting growth. 

The difference in potential between the inner and outer cell membrane is called the membrane potential and, in the resting state, membrane potential supports membrane integrity and nutrient transport [23]. In the current study, CoQ_0_ was shown to significantly hyperpolarize the membrane of *S.* Typhimurium, as shown by the negative membrane potential. We have previously shown that citral can also hyperpolarize the membrane of *Cronobacter sakazakii* [22], while membrane hyperpolarization was implicated as the antimicrobial mechanism of CoQ_0_ against *S. aureus* [19]. Similarly, polyphenols extracted from olive oil can cause cell membrane depolarization in *Cronobacter sakazakii* and *Bacillus cereus* [23,35]. Guo et al. [17] suggested that hyperpolarization or depolarization of cell membranes is caused by changes in K^+^ concentration as a result of increased membrane permeability. Therefore, we speculate that CoQ_0_ alters the permeability of the *S.* Typhimurium cell membrane, resulting in changes in cell membrane potential.

ATP is vitally important to many different physiological processes in bacteria [36]. Our results show a significant reduction (*p* < 0.01) in intracellular ATP concentration in *S.* Typhimurium cells treated with CoQ_0_. Lin, Wang, and Cui found that thyme essential oil reduced the intracellular ATP concentration of *S.* Typhimurium by 52.6% [28], and later demonstrated that the intracellular ATP concentration of *Escherichia coli* O157:H7 was reduced by 49.5% following treatment with *Litsea cubeba* essential oil [37]. The decrease in intracellular ATP concentration of *S.* Typhimurium likely results from the rapid hydrolysis of ATP or changes in cell membrane permeability.

**Figure 6:**
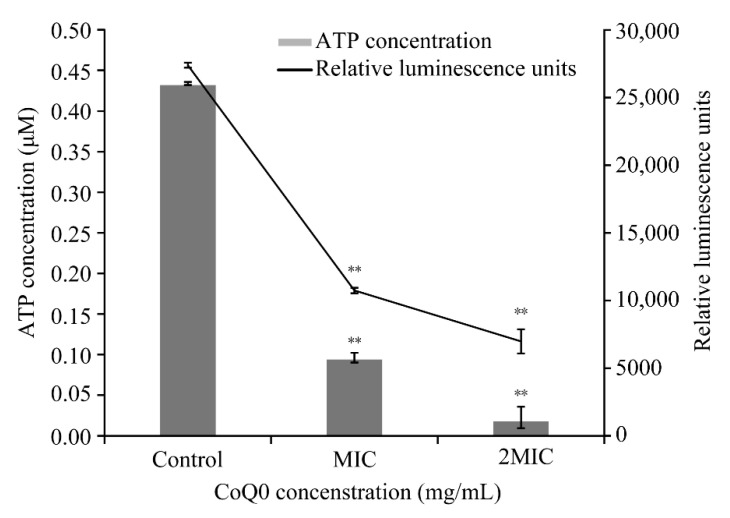
Effects of CoQ_0_ treatment on intracellular ATP concentration of *S.* Typhimurium ATCC 14028. Values represent the means of triplicate measurements. Bars represent the standard deviation (*n* = 3). Control represents no CoQ_0_ added. ** *p* < 0.01 compared with the control.

**Figure 7:**
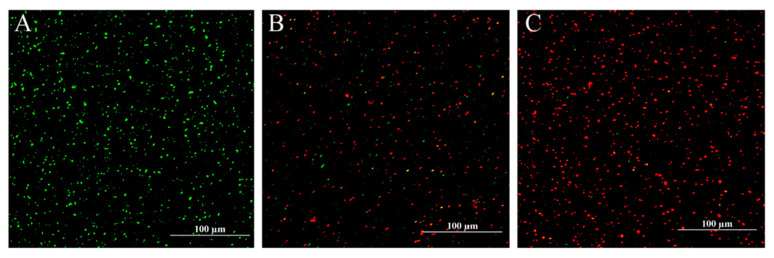
CLSM-based observation of changes in the membrane integrity of untreated *S.* Typhimurium ATCC 14028 cells (**A**) versus those treated with CoQ_0_ at MIC (**B**) and 2MIC (**C**), as demonstrated by the proportions of green versus red fluorescence.

**Figure 8:**
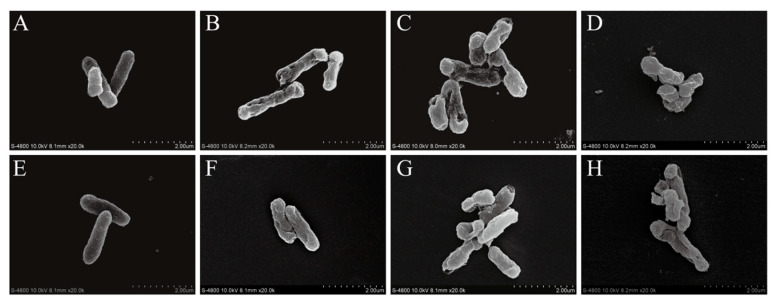
FESEM-based observations (20,000× magnification) of untreated *S.* Typhimurium ATCC 14028 at 2 (**A**) and 4 h (**E**), and of *S*. Typhimurium ATCC 14028 treated with 0.2 mg/mL CoQ_0_ for 2 (**B**) and 4 h (**F**); treated with 0.4 mg/mL CoQ_0_ for 2 (**C**) and 4 h (**G**); and treated with 0.8 mg/mL CoQ_0_ for 2 (**D**) and 4 h (**H**).

**Figure 9:**
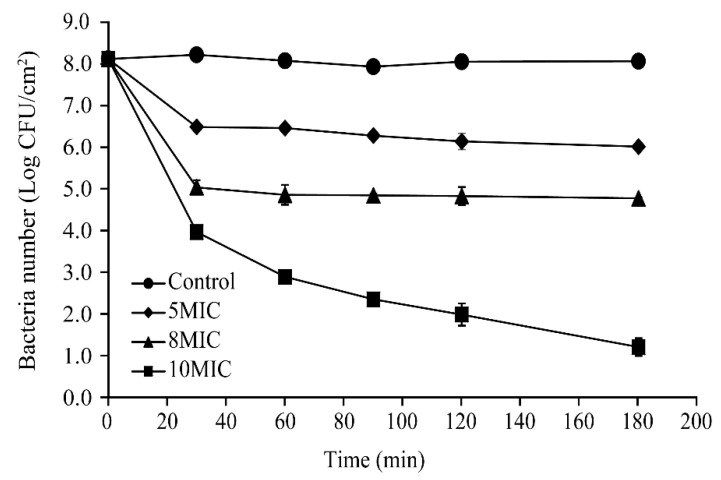
Inactivation of biofilm-associated *S.* Typhimurium ATCC 14028 cells on stainless steel surfaces following CoQ_0_ treatment. (●) Control, (♦) 5MIC, (▲) 8MIC, (∎) 10MIC. Control represents no CoQ_0_ added.

To verify the above hypothesis, we next explored the effects of CoQ_0_ on *S.* Typhimurium membrane integrity. CLSM-based analyses showed that CoQ_0_ treatment significantly reduced the integrity of *S.* Typhimurium cell membranes (Figure 6). Consistent with this observation, Fan et al. [19] and Guo et al. [17] used CLSM to show that CoQ_0_ at concentrations ranging from the MIC to 4MIC caused obvious damage to the cell membranes of *S. aureus* and *Cronobacter* *sakazakii*. FESEM analysis also showed morphological alterations to the cell membrane following CoQ_0_ treatment (Figure 7). Similarly, *Mitracarpus* *frigidus* and anthocyanins cause obvious morphological changes in the bacterial cell surface, resulting in shrinkage, corrugation, and other damage to the membranes of *S. enterica* and *S. enteritidis* cells [33,35]. Based on these observations, we propose that the morphological changes that occur in *S.* Typhimurium as a result of CoQ_0_ treatment are caused by membrane damage and permeabilization, which ultimately result in the loss of cell contents.

*S.* Typhimurium biofilms attached to various surfaces are surrounded by a matrix mainly composed of polysaccharide substances. These biofilms can cause cross-infection during food production [9]. Our study determined that CoQ_0_ at concentrations ranging from 5MIC to 10MIC caused a significant decline in the number of viable cells in *S.* Typhimurium biofilms on stainless steel surfaces. Thus, our results suggest that CoQ_0_ has the potential to control *S.* Typhimurium biofilms in the food production environment. However, because *S.* Typhimurium cells contained within a biofilm are surrounded by the matrix, it is difficult to eliminate them completely. In recent studies, Kang et al. [29] reported that a high concentration of cinnamaldehyde (18,916 μM) can make *Vibrio parahaemolyticus* in shrimp decrease to the minimum level of detection; Mohan and Purohit [38] demonstrated that pyruvic and succinic acid, and oregano essential oil had obvious antibacterial effects on planktonic *S.* Typhimurium, while high concentrations (52,000–254,000 μM) of these plant-derived antimicrobials were required to kill biofilm-associated *S.* Typhimurium. In addition, we found that conventional disinfectants have a far greater activity on the inactivation of *Salmonella* biofilms than natural substances. Byun et al. [39] found that on stainless steel surfaces, 1343 µM sodium hypochlorite and 1483 µM chlorine dioxide, respectively, reduced the number of *Salmonella* cells in the biofilm by about 4 log CFU/cm^2^ and dropped below the detection limit within 1 min. Therefore, under the premise of ensuring the good antimicrobial effect of CoQ_0_, new antibacterial technologies such as light-emitting diodes at specific wavelengths, ultrasound, and cold plasma methods could be used in combination with plant-based antimicrobials to further reduce the concentration of CoQ_0_ and effectively control food-borne pathogens and biofilms in future studies. In summary, CoQ_0_ demonstrates good bacteriostatic and bactericidal activity against *S.* Typhimurium in LB broth and raw chicken, and effectively destroyed cell membrane integrity and induced morphological changes in the cell. CoQ_0_ damages and permeabilizes the *S*. Typhimurium cell membrane, resulting in membrane hyperpolarization, decreased intracellular ATP concentration, and intracellular compound leakage. CoQ_0_ also kills biofilm-associated *S.* Typhimurium cells. Therefore, CoQ_0_ has significant potential for use as a new natural antibacterial agent for the control of *S.* Typhimurium in the food production chain, and the pharmacokinetics and toxicity of CoQ_0_ will be explored before it is used as a bacteriostatic agent.

## Figures and Tables

**Table 1:** The MICs and MBCs of CoQ_0_ against *S.* Typhimurium.

Strain	Serovar	MIC (mg/mL)	MIC (µM)	MBC (mg/mL)	MBC (µM)
ATCC 14028	Typhimurium	0.2	1098	0.2	1098
AS1.1174	Typhimurium	0.2	1098	0.2	1098
22	Typhimurium	0.2	1098	0.2	1098
63	Typhimurium	0.1	549	0.1	549
64	Typhimurium	0.2	1098	0.2	1098
80	Typhimurium	0.2	1098	0.2	1098
98	Typhimurium	0.2	1098	0.2	1098

## Data Availability

The data presented in this study are available on request from the corresponding author.
